# Microdroplet-based system for culturing of environmental microorganisms using FNAP-sort

**DOI:** 10.1038/s41598-021-88974-2

**Published:** 2021-05-04

**Authors:** Kanako Saito, Yuri Ota, Dieter M. Tourlousse, Satoko Matsukura, Hirotsugu Fujitani, Masamune Morita, Satoshi Tsuneda, Naohiro Noda

**Affiliations:** 1 Department of Life Science and Medical Bioscience, Waseda University, 2-2 Wakamatsu-cho, Shinjuku-ku, Tokyo, 162-0056 Japan; 2 Biomedical Research Institute, National Institute of Advanced Industrial Science and Technology (AIST), Center 6, 1-1-1 Higashi, Tsukuba, Ibaraki 305-8566 Japan; 3 Present Address: Faculty of Science and Engineering, Chuo University, 1-13-27 Kasuga, Bunkyo-ku, Tokyo, 112-8551 Japan

**Keywords:** Biological techniques, Microbiology

## Abstract

Droplet microfluidics has emerged as a powerful technology for improving the culturing efficiency of environmental microorganisms. However, its widespread adoption has been limited due to considerable technical challenges, especially related to identification and manipulation of individual growth-positive droplets. Here, we combined microfluidic droplet technology with on-chip “fluorescent nucleic acid probe in droplets for bacterial sorting” (FNAP-sort) for recovery of growth-positive droplets and droplet microdispensing to establish an end-to-end workflow for isolation and culturing of environmental microbes. As a proof-of-concept, we demonstrate the ability of our technique to yield high-purity cultures of rare microorganisms from a representative complex environmental microbiome. As our system employs off-the-shelf commercially available equipment, we believe that it can be readily adopted by others and may thus find widespread use toward culturing the high proportion of as-of-yet uncultured microorganisms in different biomes.

## Introduction

Even in the era of high-throughput sequencing, access to cultured isolates, that is, in vitro cultures of cells of single microbial strains, remains a prerequisite for in-depth characterization of their metabolism and physiology as well as potential biotechnological or medical applications^1^. Using standard techniques, establishing in vitro cultures for the majority of microorganisms found in diverse biomes has often proved to be ineffective. As a result, most microbes, including many phylogenetically novel groups, remain uncultured^2,3^ and thus relatively poorly understood and unexploited.

In recent years, a range of new strategies for culturing of environmental microbes have been developed to increase throughput and recover taxonomically more diverse strains. Using traditional methods that employ solid or liquid growth media for culturing of cells in bulk, overgrowth of fast-growers represents a significant hurdle impeding effective culturing of rare or slow-growing microbes. To overcome these challenges and recover a broader swath of microbes, cells can be stochastically isolated in discrete compartments prior to cultivation^4–6^. This eliminates competition for resources and inhibitory effects among microbes and has been shown to generate isolates with increased taxonomic richness, including rare and clinically relevant taxa from the human gut, compared to traditional methods^7–11^.

With the advent of microfluidic platforms that enable facile generation of thousands to millions of picoliter- to nanoliter-sized water-in-oil (w/o) droplets^12^, droplet-based cultivation strategies may thus become an important tool for cultivation-based microbiome surveys and isolation of “most-wanted” taxa from different environments^13–15^. Such systems can obtain pure cultures with high success rates by stochastically loading cells into droplets, such that the vast majority non-empty droplets will contain only a single cell, following the Poisson distribution^11,16^. However, current workflows are often difficult to adopt due to the use of custom-made microfluidic systems for droplet sorting and recovery^8,17,18^, thus necessitating further development of more accessible end-to-end workflows.

In this study, we adopted commercially available tools to develop a novel method for cultivation of environmental microbes, through combination of microfluidic droplet technology, on-chip FNAP-sort and droplet recovery using a single cell/particle type microdispenser. As previously developed by our group^19^, FNAP-sort (fluorescent nucleic acid probe in droplets for bacterial sorting) employs a fluorescence resonance energy transfer (FRET)-based RNA probe to identify droplets with growing microbial cells. In short, the RNA probe, which is added to the droplets during encapsulation of cells, is labeled with two fluorophores forming a FRET donor–acceptor pair in which fluorescence emission is quenched. During incubation, RNases are released by growing microbial cells and cleave the RNA backbone of the probe, thereby releasing the donor and acceptor fluorophores. This leads to strong fluorescence emission and thus allows identification, and sorting, of growth-positive droplets based on their increased fluorescence signal. The original FNAP-sort method was demonstrated as a technique for screening of environmental bacteria with various growth rates but did not enable recovery of the cells from individual droplets. As such, we here build on the latter work by leveraging a single cell/particle type microdispenser to recover intact droplets with viable cells suitable for scaled-up cultivation. Here, we present a proof-of-concept demonstration of the ability of our system to obtain nearly pure cultures from low-abundance microorganisms in environmental samples.

## Results and discussion

We developed and evaluated a scheme for microdroplet-based isolation and cultivation of environmental microbes using commercially available equipment for microfluidic droplet generation, on-chip fluorescence-activated cell sorting using a growth-responsive FNAP (that is, on-chip FNAP-sort)^19^, and microdispensing of growth-positive droplets with viable cells for scaled-up cultivation and identification. A general description of the workflow is discussed here, and full details are provided in the Methods section below.

**Figure 1:**
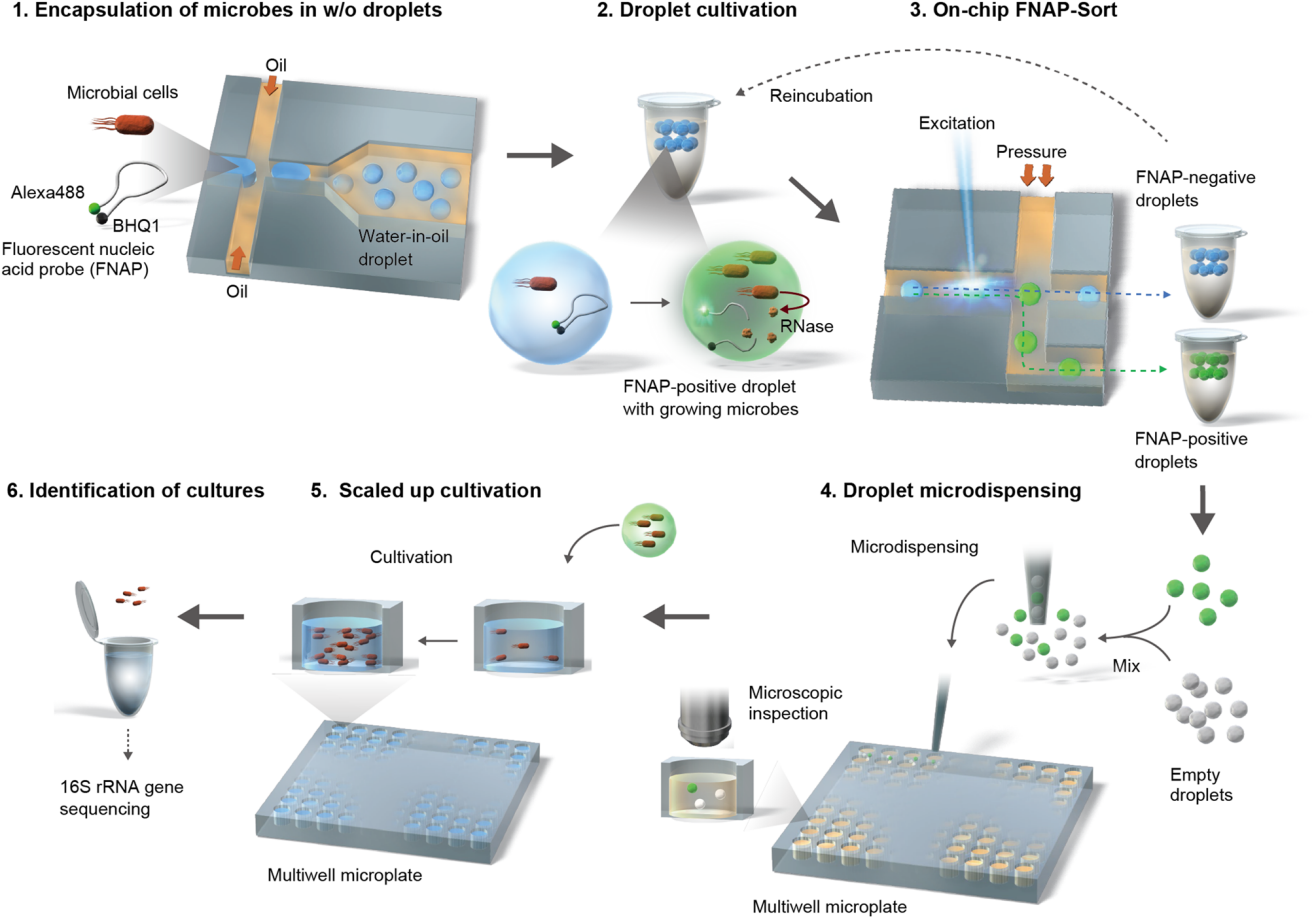
Schematic of the workflow for droplet-based cultivation and isolation of environmental microbes. Microbial cells and fluorescent nucleic acid probe (FNAP) are microfluidically encapsulated in w/o microdroplets (step 1). Following culturing under static conditions (step 2), droplets with strong FNAP-induced fluorescent signal are identified and recovered by on-chip FNAP-sort (step 3). Concurrently, droplets with weaker fluorescent signal (that is, FNAP-negative droplets) are retained and reincubated, as indicated by the dashed arrow, in order to recover slower-growing microbes. In step 4, FNAP-positive droplets are deposited in multiwell plates, following dilution with an appropriate volume of empty droplets (see text for details), by microdispensing, and optionally inspected by fluorescence microscopy. Cultures are finally scaled up (step 5) and identified by 16S rRNA gene amplicon sequencing (step 6).

As depicted in Fig. 1, the workflow starts with encapsulation of microbial cells in nanoliter-scale water-in-fluorinated-oil microdroplets containing FNAP, using a flow-focusing microfluidic droplet generator (step 1). Fluorinated oils are widely used in biological applications of droplet-based systems and are especially attractive for microbial cultivation due to their excellent biocompatibility and stability. In addition, respiratory gases, such as oxygen needed for aerobic growth, are highly soluble in fluorinated oils^20,21^. Following static incubation of the droplets (step 2), strongly fluorescent droplets (hereafter referred to as FNAP-positive droplets) are identified and recovered by on-chip FNAP-sort (step 3). To improve isolation of slower-growing microorganisms, droplets with weak fluorescence (that is, FNAP-negative droplets) are retained and further incubated; this process may be repeated multiple times depending on stability of the droplets during sorting. Subsequently, FNAP-positive droplets are deposited in multiwell microplates using an automated single cell/particle type microdispenser (step 4). Here, FNAP-positive droplets are diluted by addition of an appropriate volume of empty droplets in order to obtain, on average, single positive droplets per well. Following inspection and counting of positive droplets in each well by microscopy, the content from wells with positive droplets is then transferred to multiwell microplates containing growth medium for scaled-up cultivation (step 5). We note that microscopic counting of droplets deposited in the multiwell plates is optional. While we performed this step for development purposes, it can be omitted during routine implementation of our workflow, in order to fully leverage the strength of automated microdispensing of, stochastically, single positive droplets. Following incubation, scaled-up cultures are identified, by using *e.g.,* 16S rRNA gene amplicon sequencing (step 6).

For development and validation, we used this scheme to isolate and cultivate microbial cells from pond water as a representative environmental sample harboring highly diverse microbiota. We carefully evaluated each step in the workflow and demonstrated the feasibility of our scheme through recovery of highly pure cultures of microbes present at low abundance (< 1%) in the original inoculum.

### Microdroplet cultivation and sorting of FNAP-positive droplets

Following preparation of the inoculum (see Methods for details), microbial cells were sequestered in more than half a million of nanoliter-sized w/o droplets. Cells were then cultured under static conditions for up to 8 days and FNAP-positive droplets recovered for scaled-up cultivation and identification. More specifically, FNAP-positive droplets with proliferated microbes were recovered by on-chip FNAP-sort after 2, 5, and 8 days of culturing, with continued incubation of weakly fluorescent droplets sorted on days 2 and 5. Dark-field and fluorescence microscopy were performed to inspect the shape and size of the droplets as well as to ascertain microbial growth in the droplets.

**Figure 2:**
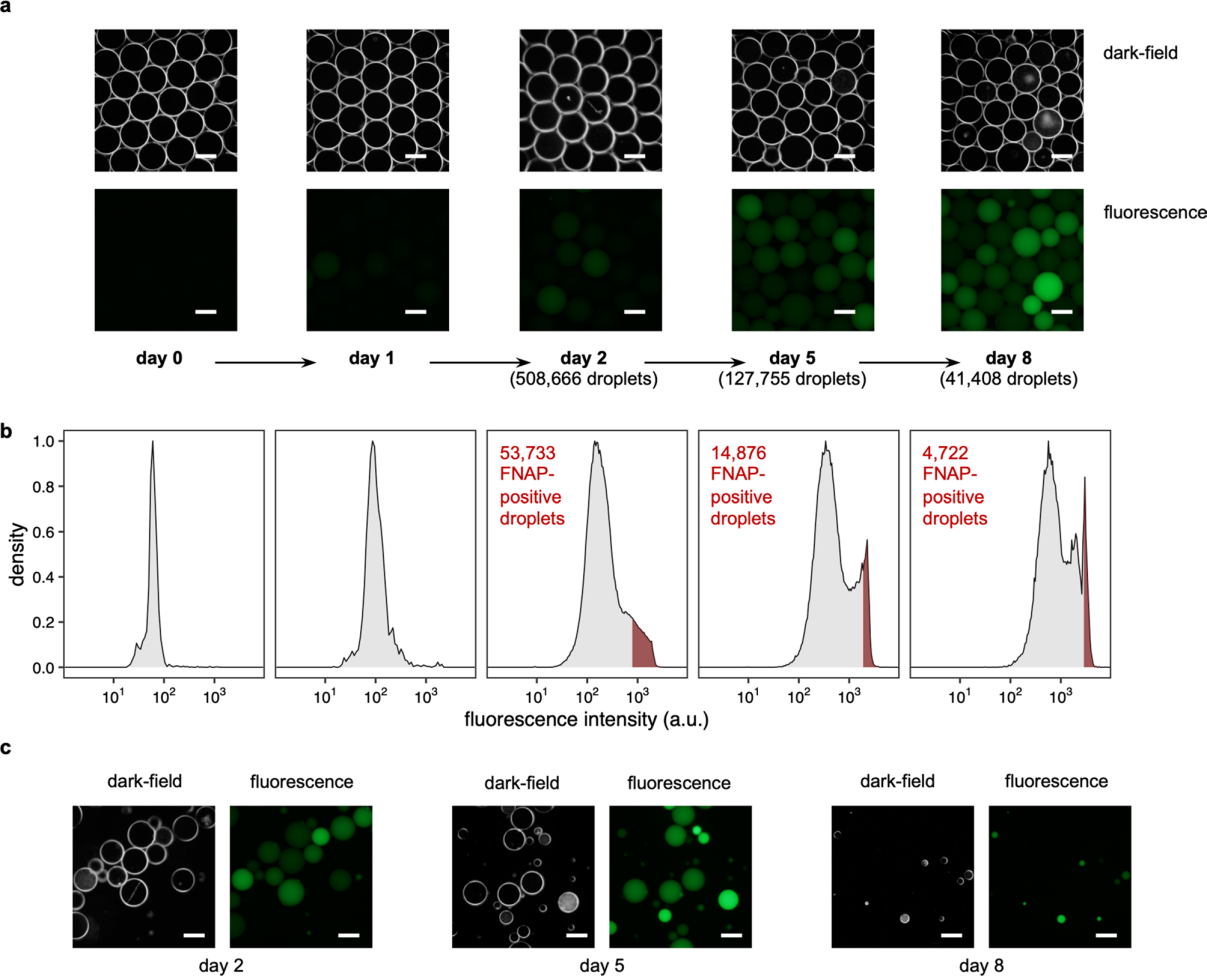
Evaluation of droplet-based cultivation and sorting. (**a**) Dark-field and fluorescence micrographs of generated droplets (day 0) and after different days of incubation (days 1–8). For days 2, 5, and 8, the total number of analyzed droplets is indicated. (**b**) Histograms of droplet fluorescence intensities as measured by on-chip flow cytometry. For days 2, 5 and 8, the red shaded area represents the top-10% brightest droplets, defined as FNAP-positives. (**c**) Dark-field and fluorescence micrographs of FNAP-positive droplets sorted after different days of incubation. The scale bar across all photographs is 100 μm.

Droplets analyzed immediately after generation (denoted as day 0) and following one day of cultivation (day 1) were uniform in size, with a diameter of approximately 120 μm, and displayed no visible fluorescence by microscopy (Fig. 2a). Fluorescence intensities of approximately 3,000 droplets measured by on-chip flow cytometry were narrowly distributed (Fig. 2b) but were slightly shifted toward higher intensities on day 1. This shift may be attributed to several processes, such as activity of ribonucleases present in the growth medium, non-enzymatic degradation of the RNA backbone of the probe and instability of the quenching dye.

After the second day of incubation, microscopic inspection showed increased fluorescence in a portion of droplets as well as visible small clusters of cells in some droplets by dark-field microscopy (Fig. 2a). We thus proceeded with sorting of the droplets by on-chip FNAP-sort, as follows. To determine a fluorescence intensity threshold for delineating FNAP-positive droplets, we sacrificed a few thousand droplets for measuring their fluorescence intensity by flow cytometry. This showed that the distribution of droplet fluorescence intensities became right-tailed (Fig. 2b), suggesting that microbial growth had occurred in a considerable fraction of droplets. At this stage, we used the 90% percentile of measured intensities as cutoff for gating, thus retaining the top-10% brightest droplets for scaled-up cultivation (see next section). This strategy, rather than using a hard fluorescence intensity threshold, was chosen to accommodate the continued shift of the distribution toward higher intensities over time, as discussed above. To recover slower-growing microbes, droplets with fluorescence intensity below the threshold were retained and further incubated.

Microscopic inspection of the latter droplets after three days of additional incubation (denoted as day 5, the total incubation time) revealed increased fluorescence as well as visible growth by dark-field microscopy (Fig. 2a). Fluorescence intensities measured by flow cytometry became bimodal, with a clear high-intensity peak (Fig. 2b). Again, we retained the top-10% brightest droplets by on-chip FNAP-sort and re-incubated the remaining droplets for an additional three days. Flow cytometric analysis showed a multimodal distribution of fluorescence intensities, indicating that multiple discrete populations of droplets containing cells with varying growth characteristics had emerged on day 8. Droplet size and uniformity of FNAP-positive droplets after sorting on day 8 were however substantially reduced (Fig. 2c). We thus retained FNAP-positive droplets after 2 and 5 total days of incubation for further analysis. The reason for the increased droplet heterogeneity on day 8 remained unclear but was presumed to be due to merging or splitting of droplets and/or evaporation upon repeated sorting. To alleviate such issues, it is possible to continuously incubate the droplets for extended periods of time, without repeated sorting to recover microorganisms with different growth rates.

### Collection and processing of FNAP-positive droplets by microdispensing

Following FNAP-sort, positive droplets were deposited in 384-multiwell microplates using the On-chip SPiS instrument. Because the image recognition technology used by the latter system for retrieval of single particles was ineffective for w/o droplets due to their natural buoyancy that interfered with the particle detection algorithm, we performed a preliminary analysis to experimentally determine the number of droplets deposited per well, for the specific operation conditions used (see Methods for details). Using fluorescence microscopy for droplet counting, this showed that an average of three to four droplets were deposited per well (data not shown). As such, we diluted the FNAP-positive droplets by addition of empty droplets in order to ensure that each well will, on average, receive a single FNAP-positive droplet and two empty droplets (see step 4 in Fig. 1).

**Figure 3:**
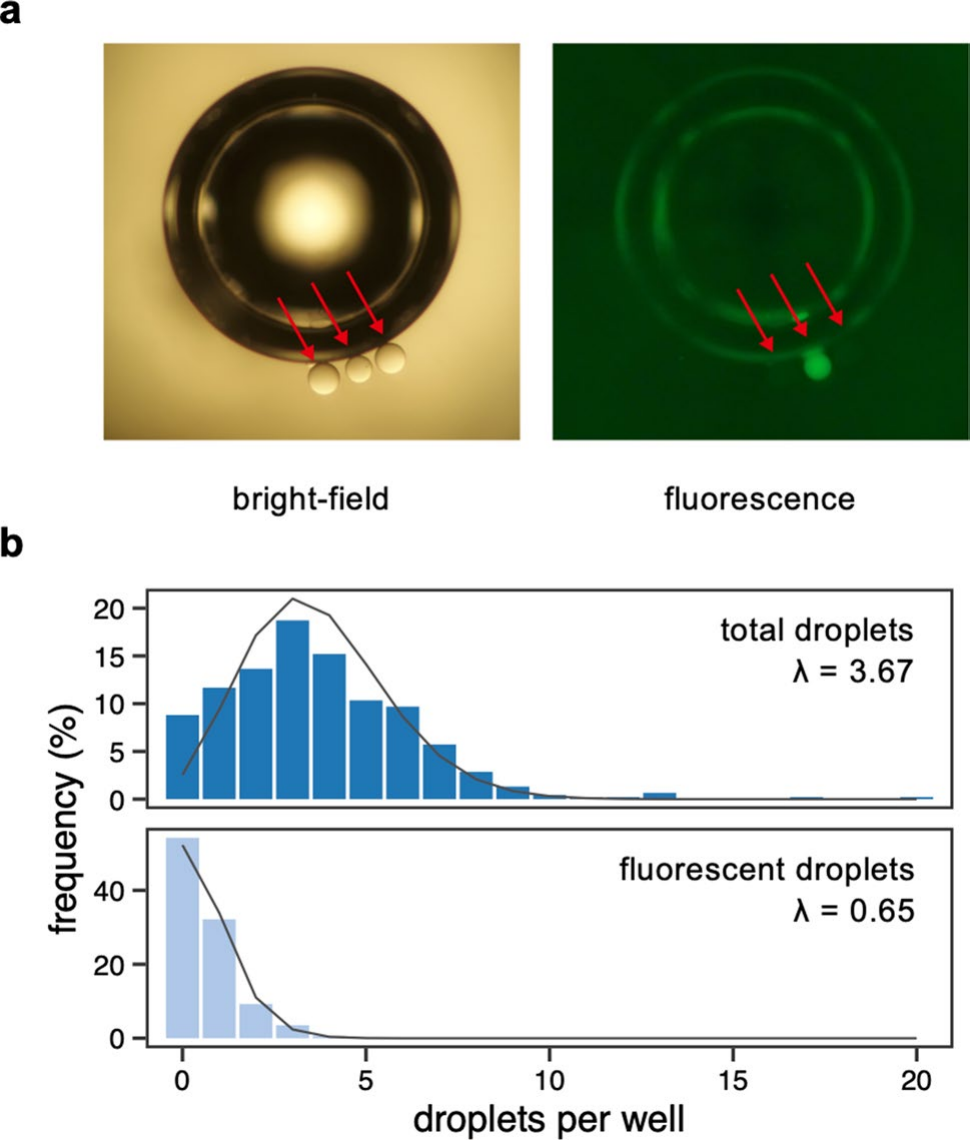
Droplet collection by microdispensing. (**a**) Bright-field (left panel) and fluorescence (right panel) micrographs of a single representative well containing three total droplets (marked by arrows), with a single fluorescent droplet in the middle. Note that the large concentric circles visible in both images are due to an air bubble entrapped in the microwell. The diameter of droplets is approximately 120 µm. (**b**) Frequency of total and fluorescent (that is, FNAP-positive) droplets collected in multiwell microplates by microdispensing, as evaluated by bright-field and fluorescence microscopy. Grey solid lines represent Poisson distributions fitted to the data. Indicated lambda (λ) values are the expected number of droplets per well based on the fitted Poisson distributions.

Using this approach, FNAP-positive droplets, after dilution, collected on days 2 and 5 were transferred to at least 200 wells (namely 215 and 239 for days 2 and 5, respectively) of a 384-well microplate, respectively. For this proof-of-concept study, this was sufficient to yield two 96 wells for downstream processing (that is, scaled-up cultivation as described below). The multiwell plates were preloaded with fluorinated oil (with fluorosurfactant) and mineral oil. Addition of mineral oil, located on top, led to a convex meniscus at the interphase with the fluorinated oil layer. As a result, the buoyant droplets migrated towards the center of the well, allowing for easy visualization (Fig. 3a). For the 454 wells evaluated, this showed that 146 wells (32.2%) contained a single FNAP-positive fluorescent droplet, 42 wells (9.3%) contained two positive droplets and 20 wells (4.4%) contained three or more positive droplets (Fig. 3b).

We note that because in our technique multiple droplets are collected per well, each containing on average a single positive droplet after appropriate dilution with an emulsion of empty droplets, alternative low volume dispensers could potentially also be used. Nevertheless, in our hands, the On-chip SPiS instrument was useful as it allowed careful control of droplet collection performance, by setting *e.g.,* the speed of vertical movement of the pipette tip in the emulsion, as detailed in the Methods section. Still, although the throughput of droplet collection may be readily increased, we noticed that upon repeated dispensing, the number of collected droplets may slightly change because of the reduced thickness of the remaining droplet layer in the fluorinated oil.

### Scaled-up cultivation and sequencing-based identification of cultures

We then transferred the content of the wells with one or two FNAP-positive droplets to 96-well plates, prefilled with 160 μl of R2A medium, for scaled-up cultivation. Here, the R2A medium destabilizes the droplets, leading to effective release of the microbial cells by repeated pipetting. Following cultivation for about two weeks under static conditions, growth in the wells was evaluated by phase-contrast microscopy and optical density measurements. Based on this, we selected a total of 21 scaled-up cultures for identification by 16S rRNA gene amplicon sequencing. To this end, we performed direct-PCR amplification of the V4 hypervariable region, which resulted in successful amplification for 15 of the cultures. Lack of PCR amplification for the remaining cultures may be due to inhibition or mismatches to the PCR primers.

**Figure 4:**
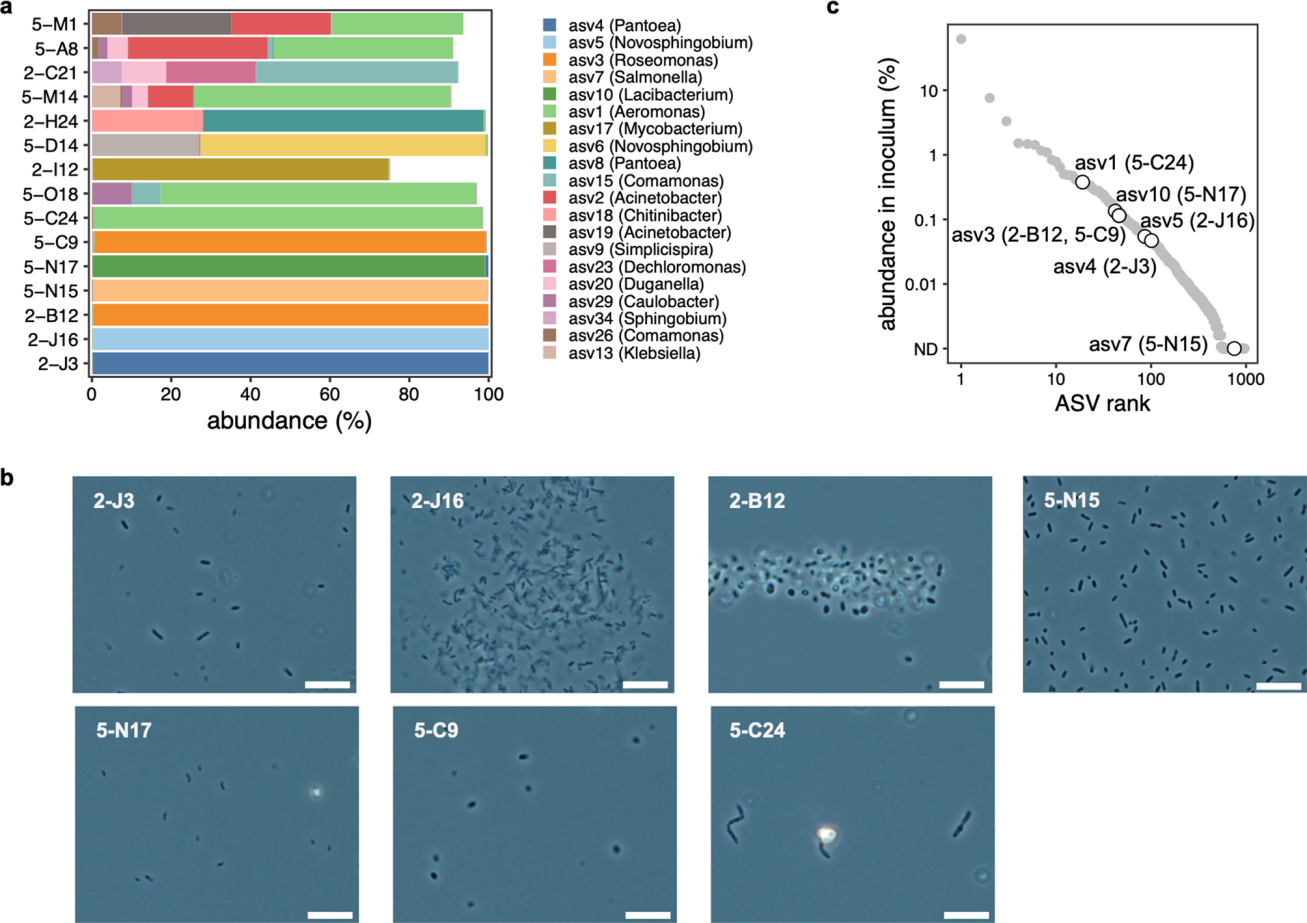
Screening and identification of scaled-up cultures. (**a**) Bar charts showing the composition of the generated cultures, as evaluated by 16S rRNA gene amplicon sequencing. The top-20 most abundant ASVs, based on maximum abundance across cultures, are shown and cultures are sorted along with the y-axis based on the abundance of the most dominant ASV. Fill colors represent ASVs, with corresponding genus-level taxonomic assignments shown in the legend. Culture identifiers shown on the *y*-axis reflect the incubation period and well number of the scaled-up culture multiwell plate, separated by a hyphen (for example, culture 2-J3 was obtained after two days of cultivation in droplets and cultured in well J3). (**b**) Phase contrast images of cells in scaled-up cultures containing a single dominant ASV with an abundance of at least 98%. The scale bar for all photographs is 10 µm. (**c**) Rank abundance curve of ASVs in the source sample (that is, water from an artificial pond). Circles represent ASVs recovered in scaled-up cultures containing a single dominant ASV with an abundance of at least 98%, with corresponding culture identifiers indicated between brackets. ND: not detected.

Following sequencing on a MiSeq instrument (2 × 251 bp reads), amplicon sequence variants (ASVs) were reconstructed using DADA2 for taxonomic identification of the cells in the cultures (Supplementary Table S1). As shown in Fig. 4a, seven of the cultures were dominated by a single ASV with an abundance of at least 98%. These cultures were thus presumed to be nearly pure and may be readily further purified using traditional techniques. Here, we recognize however that closely related strains or species may not be distinguishable based on short 16S rRNA gene amplicon sequences. Remaining sequencing reads for the high-purity cultures were distributed across multiple rare ASVs (Supplementary Fig. S1). Based on their low sequence similarity to the main ASV and/or low abundance, these ASVs were probably derived from nonviable cells or relic DNA of death cells that is carried across the workflow or minor live contaminants in the cultures. The other cultures were mixed, with abundant ASVs belonging to varying genera (Fig. 4a, Supplementary Fig. S2).

As shown in Fig. 4a, the high-purity cultures represented microbes that are prevalent in environmental fresh waters. These included members of the genera *Pantoea* (asv4 in culture 2-J3 containing rod-shaped cells as shown in Fig. 4b), *Novosphingobium* (asv5 in culture 2-J16, rod-shaped), *Roseomonas* (asv3 in cultures 2-B12 and 5-C9, coccoid-shaped), *Lacibacterium* (asv10 in culture 5-N17, small rod-shaped) and *Aeromonas* (asv1 in culture 5-C24, rod-shaped). These data verified that our workflow recovered isolates that are of biological relevance to the studied ecosystem. We note that asv7 in culture 5-N15 was not detected in the inoculum at the evaluated sequencing depth; it may thus represent a very low-abundance species, although it cannot be excluded that it is a contaminant introduced during the workflow. Further, ASVs in the nearly pure cultures were relatively rare in inoculum. More specifically, as shown as a rank abundance curve in Fig. 4c, ASVs in the highly pure cultures had a relative abundance of less than 1% in the inoculum. This demonstrated that by single droplet isolation, we were thus able to obtain rare microbes from complex microbiota.

## Conclusion

To summarize, we established and evaluated a workflow for isolation and cultivation of environmental microbes, by combining microfluidic droplet generation with on-chip FNAP-sort and microdispensing for recovery and scaled-up cultivation of growth-positive droplets. Using microbes for a diverse environmental microbiome, we showed that this system can generate nearly pure cultures of microbes present at low abundances in the original sample. To improve throughput, the number of scaled-up cultures can be increased, as this represented the main bottleneck in our proof-of-concept study. Importantly, as our system is based fully on commercially available off-the-shelf equipment, it should be readily adoptable by other researchers and may thus find widespread use for isolation of as-of-yet uncultured microorganisms in different biomes.

## Methods

### Preparation of inoculum from environmental sample

Water sample was obtained from an artificial pond at the premises of the National Institute of Advanced Industrial Science and Technology (AIST), Japan, in November 2019. A grab sample was collected in a 2.2-l autoclaved plastic bottle, roughly 5 cm below the surface of the water. To remove large particles and debris, the water sample was passed sequentially through a 41-µm and a 20-µm nylon net filter (Merck Millipore, Billerica, MA, USA). The filtered sample was then centrifuged at 12,000 × *g* for 20 min at 18 °C and 400 ml of subnatant with cell pellet recovered. The latter was centrifuged (9,510 × *g* for 20 min at 18 °C) and the recovered cell pellet washed with R2A medium (bacto yeast extract, 0.5 g/l; bacto proteose peptone No. 3, 0.5 g/l; casamino acids, 0.5 g/l; glucose, 0.5 g/l; soluble starch, 0.5 g/l; K_2_HPO_4_, 0.3 g/l; MgSO_4_⋅7H_2_O, 0.05 g/l; sodium pyruvate, 0.3 g/l). The washing step was repeated two times and cells were finally resuspended in 17 ml of R2A medium. Cells were stained with SYTO 9 Green Fluorescent Nucleic Acid Stain (Thermo Fisher Scientific, Waltham, MA, USA) and counted by fluorescence microscopy.

### Microfluidic droplet generation and incubation

Cell suspension of environmental microbes as prepared above was diluted in R2A medium to a concentration of approximately 10^7^ cells per ml; FNAP (5′-Alexa488-UCUCGGUGCGUUG-BHQ1-3′) was added to a final concentration of 200 nM^19^. Water-in-oil (w/o) droplets were generated with a QX100 droplet generator (BioRad, Hercules, CA, USA) using DG8 cartridges. The oil phase consisted of Novec 7500 Engineered Fluid fluorinated oil (NT Science, Nagoya, Japan) and 2% of 008-FluoroSurfactant (w/w, On-chip Biotechnologies, Tokyo, Japan). Generated droplets (~ 500 µl in total) were collected in 1.5 ml tubes. Incubation was performed in the dark at 18 °C under static conditions.

### Microscopy and optical density measurements

Droplet emulsions were loaded into a µ-Slide VI flat 6-channel microslide (ibidi GmbH, Martinsried, Germany) prefilled with fluorinated oil (Novec 7500 oil with 2% of 008-FluoroSurfactant). Microbial cells were deposited onto plain glass microscope slides. Dark-field and fluorescence images of droplets and phase contrast images of bacterial cells were captured with an Axio Imager 2 (Carl Zeiss, Jena, Germany) equipped with a DP72 camera (Olympus, Tokyo, Japan) operated by DP2-BSW software (Olympus). Bright-field and fluorescence images of droplets in 384-multiwell microplate were obtained using a Power IX71 (Olympus) equipped with a STYLUS 1 s digital camera (Olympus). Optical density of cultures in 96-multiwell microplates was measured using a DTX 880 Multimode Detector (Beckman Coulter, Brea, CA, USA).

### Measurements of fluorescence intensity of droplets and droplet sorting

Droplets were analyzed using an On-chip Sort instrument (On-chip Sort MS5G; On-chip Biotechnologies), equipped with a 488-nm excitation laser, a photodiode for forward-scatter detection, photomultiplier tube for green fluorescence detection (543 ± 22 nm) and OnChipFlow software (version 1.9.16). Both sample and sheath pressure were set to 0.12 psi. A 150-μm channel microfluidic chip (2D Chip-Z1000-w150; On-chip Biotechnologies) was used, containing 8 ml of Novec 7500 oil with 0.01% 008-FluoroSurfactant in the sheath reservoir, 1.5 ml of Novec 7500 oil with 0.01% 008-FluoroSurfactant in the sorting reservoir, and 100 µl of Novec 7500 oil with 2% 008-FluoroSurfactant in the collection reservoir.

For sorting, a fluorescence threshold for gating was determined by measuring the fluorescence intensity of ~ 3,000 sacrificed droplets and set to the 90% percentile of measured intensities. Droplets with a fluorescence intensity above this cutoff were classified as FNAP-positive droplets and deflected to the collection reservoir. If applicable, unsorted droplets with weak fluorescence signal (here referred to as FNAP-negative droplets) were collected from the waste reservoir and transferred to a clean 1.5-ml tube for further incubation.

### Droplet microdispensing

Following sorting of FNAP-positive droplets, microdispensing of the droplets into multiwell microplates was performed using an On-chip SPiS instrument (On-chip Biotechnologies), with On-chip SPiS software (version 204–1) and KV Studio (version 8, Keyence, Osaka, Japan). To this end, droplets (5 µl, after dilution with an emulsion of empty droplets, see Discussion for details) were placed in a 0.2-ml tube containing 100 µl of Novec 7500 oil with 2% 008-FluoroSurfactant and 150 µl of mineral oil (Sigma-Aldrich, St. Louis, MO, USA). The filled 0.2-ml tube, extra-fine dispensing tips (Chip-384S, On-chip Biotechnologies), 300-µl low-retention filter tips (Greiner Bio-One, Kremsmünster, Austria) for liquid level detection and mixing, and a 384-multiwell microplate were placed into the On-chip SPiS device. Small sample aliquots were aspirated from the 0.2-ml tube and dispensed into individual wells of a 384-well microplate, prefilled with 20 µl of Novec 7500 oil with 0.01% 008-FluoroSurfactant and 20 µl of mineral oil per well. Note that automatic image recognition for single cell/particle detection was deactivated as preliminary experiments proved that this was ineffective for w/o droplets due to their buoyancy.

Default settings of the On-chip SPiS instrument control software were used for droplet collection, except for the KV Studio parameter DM6290. This parameter controls the speed at which the pipette is withdrawn from the sample and was set to 35 based on preliminary experiments.

The mode of droplet collection using the On-chip SPiS instrument, as adopted here, was as follows. The droplets were present in a small vessel (0.2-ml tube) containing fluorinated oil (with fluorosurfactant) and mineral oil, resulting in a phase of fluorinated oil at the bottom, a layer of droplets (which are buoyant in the fluorinated oil) in the middle, and a phase of mineral oil at the top. Here, the mineral oil was included such that the fluorinated oil with buoyant droplets formed a convex meniscus at the interphase with the mineral oil, as a result of which the buoyant droplets migrated towards the center of the well. Upon sampling, the extra-fine pipette tip used by the On-chip SPiS instrument was lowered through the different layers, namely the mineral oil phase, droplet emulsion, and fluorinated oil phase. As the pipette tip passed through the droplet layer, droplets were taken up in the pipette tip due to capillary action. Once the pipette tip reached the fluorinated oil phase, a small amount of oil was aspirated. Finally, the pipette tip was withdrawn from the tube, for dispensing of the collected sample in a multiwell plate. As the pipette tip moved through the droplet layer, additional droplets were again drawn into the tip. As such, the number of collected droplets depended on several factors, mainly the height of the droplet layer and the speed of vertical movement of the pipette tip through the droplet layer. Based on trial-and-error, we found that using a relatively small droplet volume (5 µl), using mineral oil at the top, and optimizing the speed at which the pipette was withdrawn vertically from the tube (parameter DM6290, see above), resulted in reproducible collection of approximately three droplets per sample.

### Scaled-up cultivation from microdispensed droplets

Following microscopic inspection of the microdispensed droplets, droplets were transferred from the 384-well plate to 96-well plates, prefilled with 160 µl of R2A medium per well. Droplets were disrupted by repeated pipetting following transfer to ensure complete release of the microbial cells from the droplets. Incubation was performed at 18 °C without shaking. Plates containing droplets sorted on days 2 and 5 of droplet cultivation were incubated for 14 and 12 days, respectively.

### DNA extraction

Extraction of DNA from the concentrated cell suspension prepared from the pond water sample (see “Preparation of environmental sample”) was performed using the FastDNA SPIN Kit for Soil (MP Biomedicals, Santa Ana, CA, USA). After centrifugation at 10,000 × *g* for 3 min at 18 °C, the cell pellet was resuspended with 978 µl of Sodium Phosphate Buffer and 122 µl of MT Buffer, both part of the kit. All subsequent steps followed manufacturer’s recommended procedures and purified DNA was finally obtained in 100 µl of ultra-pure DNase/RNase-free water. For the scaled-up cultures, 150 µl of culture liquid was centrifuged at 12,000 × *g* for 3 min and supernatants discarded. Pelleted cells were then resuspended in 30 µl of 10 mM Tris–HCl (pH 8.0), transferred to 0.2 µl tube and heated for 5 min to 95 °C for cell lysis. Heated-treated cells were subjected directly to PCR amplification without purification, as described below.

### Construction of 16S rRNA gene library and sequencing

Libraries of 16S rRNA gene amplicons were prepared as described previously^19^, with minor modification. More specifically, the V4 hypervariable region of the 16S rRNA gene was PCR amplified using forward primer 515F (5′-GTGCCAGCMGCCGCGGTAA-3′) and Golay-barcoded reverse primer 806R (5′-GGACTACHVGGGTWTCTAAT-3′)^22^. Primers were obtained from Tsukuba Oligo Service (Tsukuba, Japan). PCR reactions (20 µl) contained 1 × PCR Buffer I with MgCl_2_, 200 µM of each dNTP (both from Thermo Fisher Scientific), 200 nM of forward and reverse primers each, 1.25 units of AmpliTaq Gold DNA Polymerase (Thermo Fisher Scientific), and 1 µl of sample (that is, heat-lysed cell suspension of the scaled-up cultures or ~ 20 ng of environmental DNA). Thermal cycling conditions consisted of enzyme activation for 5 min at 94 °C, followed by 35 cycles of 94 °C for 30 s, 50 °C for 30 s, and 72 °C for 1 min, and a final 5 min extension step at 72 °C. Amplicon from three replicates were combined and purified using the Agencourt AMPure XP System (Beckman Coulter). Purified libraries were quantified using the Agilent DNA 1000 kit (Agilent Technologies, Santa Clara, CA, USA) and Agilent Bioanalyzer 2100 system (Agilent Technologies) and pooled at equimolar concentration. Pooled libraries were supplemented with 50% PhiX DNA and sequenced on a MiSeq instrument (Illumina, San Diego, CA, USA), using v2 chemistry (2 × 251-bp reads).

### Reconstruction amplicon sequencing variants

Amplicon sequence variants (ASVs) were generated using DADA2 v1.16^23^. In short, reads were trimmed and filtered with parameters truncLen = c(200,150), maxEE = c(2,4) and maxN = c(0,0). All subsequent steps (that is, error model learning, denoising, merging of denoised reads and bimera filtering) were performed with default settings, except that 3 × 10^8^ bases were used for error model learning. Taxonomy was assigned to the ASVs using DADA2′s assignTaxonomy function against the RDP trainset 18 (release 11.5)^24^, with a default minimum bootstrap confidence (minBoot) of 50. A final ASV table was generated by eliminating ASVs with unexpected size (< 250 bp or > 255 bp) or lacking taxonomy assignment at the phylum level.

### Data analysis

All data were imported into R (v4.0.2)^25^, and packages dplyr v1.0.2^26^ and ggplot2 v3.3.2^27^ were used for data manipulation and visualization, respectively. Parameter estimation of the Poisson distribution of droplet counts was performed using the function fitdist in the R package fitdistrplus v1.1^28^. Bin densities, scaled to maximum of 1, for the fluorescence intensity histograms were calculated using ggplot2′s geom_histogram function.

## Supplementary Information

Supplementary Information

## Data Availability

All raw sequencing data have been deposited in NCBI’s Sequence Read Archive repository under BioProject PRJNA699411.
