# Supplementary material for: Microdroplet-based system for culturing of environmental microorganisms using FNAP-sort
Source: Sci Rep. 2021 May 4;11:9506. doi: 10.1038/s41598-021-88974-2 (PMC8096817; doi:10.1038/s41598-021-88974-2)
Supplement: Supplementary file 1 — Supplementary Information [file 41598_2021_88974_MOESM1_ESM.pdf]

Microdroplet-based system for culturing of environmental microorganisms using FNAP-sort

Kanako Saito, Yuri Ota, Dieter M. Tourlousse, Satoko Matsukura, Hirotsugu Fujitani, Masamune Morita, Satoshi Tsuneda & Naohiro Noda

Supplementary Table S1. Summary statistics of sequencing data.

sample ID	input read pairs	filtered and trimmed read pairs	denoised forward reads	denoised reverse reads	merged read pairs	non-bimeric	accession number
inoculum	241468	216669	214037	209884	201514	195042	SAMN17775858
2-B12	226989	208052	207929	202804	199893	199879	SAMN17775859
5-C24	222372	193163	192983	189501	183761	182904	SAMN17775860
5-N17	176619	163938	163784	161479	159736	159557	SAMN17775861
5-C9	239389	201026	200848	195835	191570	191542	SAMN17775862
2-C21	166006	150966	150814	145200	141342	136512	SAMN17775863
2-J16	238214	224238	224146	218576	216590	216590	SAMN17775864
2-J3	246597	228881	228795	225340	223155	223155	SAMN17775865
2-H24	310990	273150	272841	262653	251719	219607	SAMN17775866
2-I12	125704	111270	110588	107339	105276	104333	SAMN17775867
5-M1	119556	109606	109421	107138	104933	96226	SAMN17775868
5-N15	247491	213939	213789	207470	199562	199543	SAMN17775869
5-A8	163522	147348	147209	140380	136176	130907	SAMN17775870
5-D14	346695	305263	305060	291418	283512	281137	SAMN17775871
5-M14	170256	155198	155040	152231	147879	137609	SAMN17775872
5-O18	126783	113954	113838	110347	107526	101087	SAMN17775873

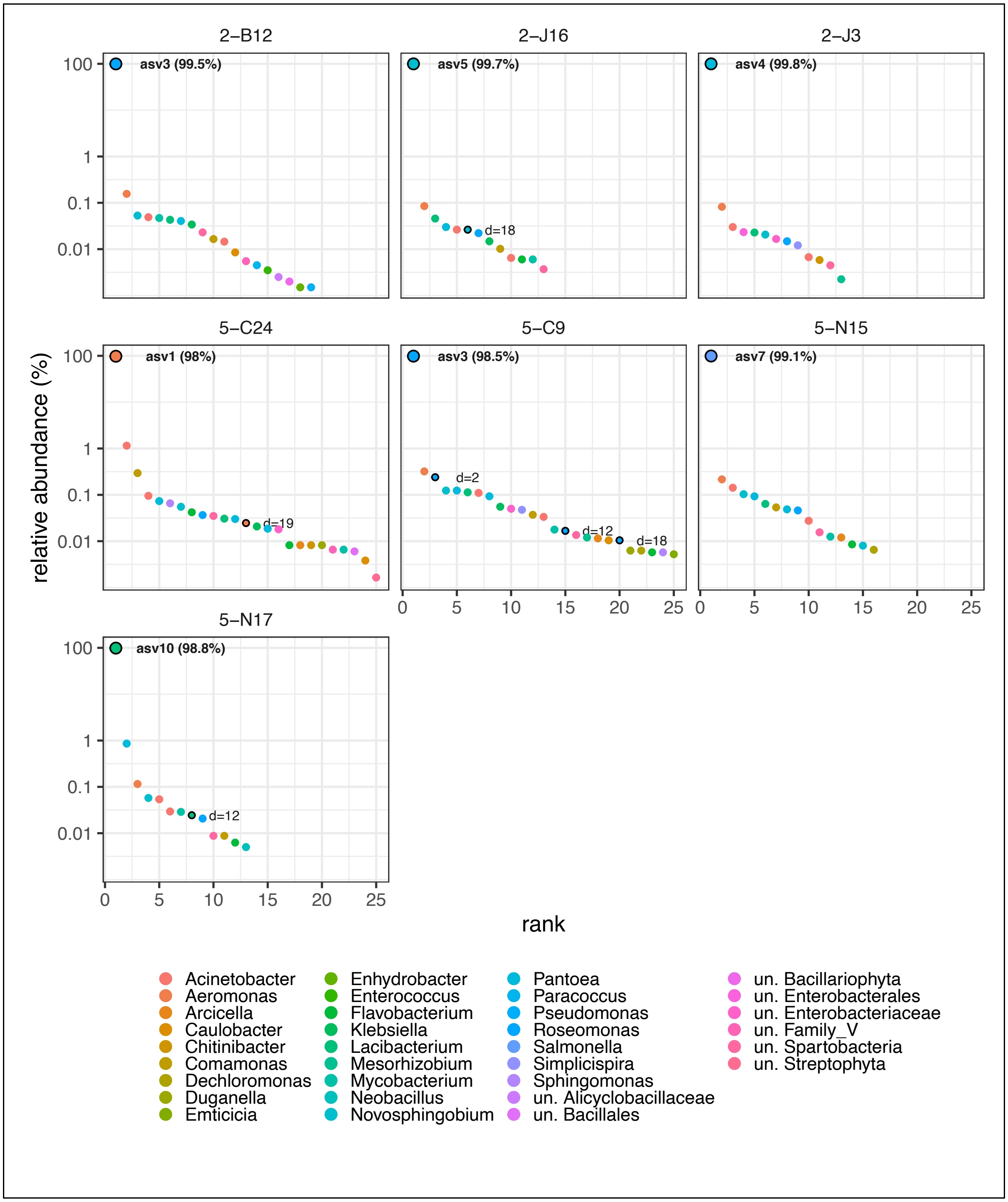

Supplementary Figure S1. Rank abundance curves for ASVs in the high-purity cultures, that is cultures with a dominant ASV with an abundance of ≥98%. Fill colors represent genus-level taxonomic assignments. Edit distances, calculated using the function stringdist in the R package stringdist v0.9.6.3 with method="lv", of individual low-abundance ASVs (marked by a solid line) to the main ASV for each cultures are indicated ("d=") in case of matching genus-level taxonomic assignments for the dominant and low-abundance ASVs.

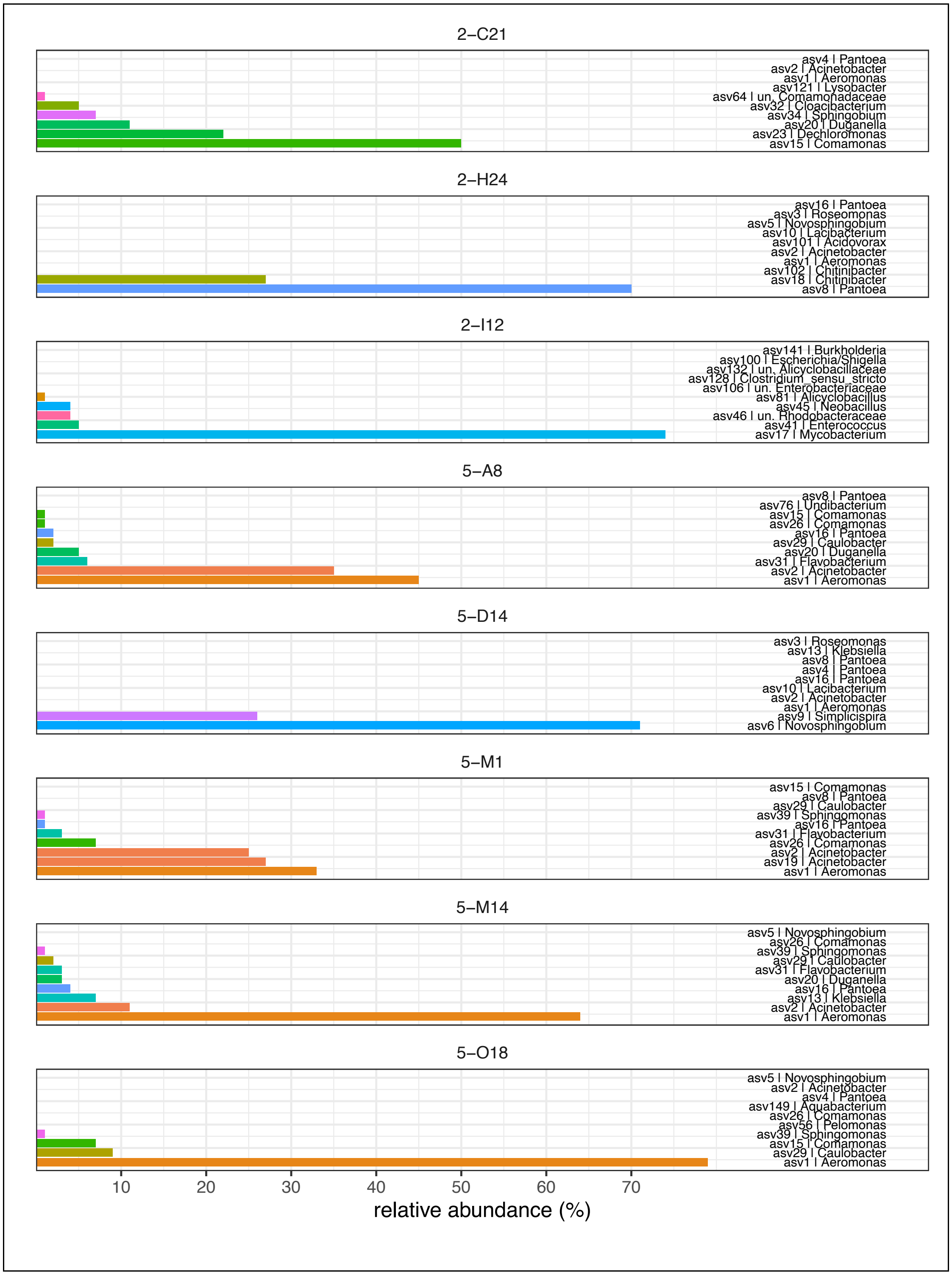

Supplementary Figure S2. Barcharts of the abundance of the top-10 most abundant ASVs in each of the low-purity cultures. Fill colors represent genus-level taxonomic assignments.
